# Analytical Investigation of Primary Waveform Distortion Effect on Magnetic Flux Density in the Magnetic Core of Inductive Current Transformer and Its Transformation Accuracy

**DOI:** 10.3390/s25154837

**Published:** 2025-08-06

**Authors:** Michal Kaczmarek, Kacper Blus

**Affiliations:** Institute of Mechatronics and Information Systems, Lodz University of Technology, 90-537 Lodz, Poland; kacper.blus@dokt.p.lodz.pl

**Keywords:** inductive current transformer, distorted waveform, RMS values of low-order higher harmonics, resulting magnetic flux density, influence of phase angles of higher harmonics, transformation accuracy

## Abstract

This paper analyzes how distortion in the primary current waveform affects the magnetic flux density in the magnetic core of an inductive current transformer and its transformation accuracy. Keeping the primary current’s RMS value constant, it studies the impact of changes in the RMS values and phase angles of low-order harmonics on the core’s flux density and the values of current error and phase displacement of their transformation. The distorted current waveforms, resulting flux density, and hysteresis loops are examined to identify the operating conditions of the inductive current transformer. This study also highlights the strong influence of low-order harmonics and the diminishing effect of higher-frequency harmonics on the magnetic flux density in its magnetic core, e.g., third, fifth, and seventh higher harmonics may cause an increase in magnetic flux density in the magnetic core of the inductive current transformer in relation to that obtained for a sinusoidal current with a frequency of 50 Hz by about 8.5%, while with additional second, fourth, and sixth harmonics, the increase may reach about 23%. Therefore, the testing procedure should consider not only the load impedance and the RMS values of the primary current but also its harmonic content, including the RMS values of individual harmonics and their phase angles.

## 1. Introduction

Wideband transformation accuracy of an inductive current transformer (iCT) for metering purposes is required for the measurement of distorted electrical power and assessment of the power quality. This is due to the fact that modern electrical power systems supply nonlinear loads such as variable frequency drives, LED lighting, and power electronic converters, which produce significant values of higher harmonics into the current. The third harmonic is caused by the drawing of current in pulses and the operation of inductive devices, while the six-pulse rectifiers (common in industrial power converters) produce 5th, 7th, 11th, 13th harmonics. Moreover, the negative influence on the power quality of renewable energy sources [1,2,3,4,5] is also important. Previous research on inductive current transformers (iCTs) has largely focused on their performance under sinusoidal conditions, with primary attention given to load impedance, RMS current levels, and thermal behavior. While some studies have addressed the general influence of harmonic distortion on transformer operation, they typically emphasize power quality or overall system performance rather than the specific internal magnetic behavior of iCTs. Notably, little to no prior work has systematically investigated how variations in the RMS values and phase angles of individual low-order harmonics affect the magnetic flux density within the core and the associated measurement errors (e.g., current error and phase displacement). In most existing standards and testing procedures, the harmonic content of the primary current is either neglected or treated as a secondary factor, with minimal attention paid to its influence on flux waveform distortion and core saturation dynamics. This gap indicates that the detailed magnetic response of iCTs to distorted current waveforms—particularly the contribution of specific harmonics to flux distortion and hysteresis behavior—has not been adequately analyzed or quantified. In the paper in [6], a key finding is that the harmonic power error increases with the absolute magnitude of the iCT phase displacement. Specialized measuring systems have been developed to assess the wideband transformation accuracy of iCTs under distorted current conditions. These systems are designed to evaluate their ability to accurately reproduce current signals across a broad frequency range, particularly when the input current includes harmonic components or other forms of waveform distortion often present in modern electrical networks [7,8,9,10,11,12,13,14]. The paper in [7] underscores the need to evaluate instrument transformers under realistic, combined influence conditions to ensure accurate performance in modern, distorted power systems. The study used wideband distorted waveforms combining fundamental frequency, harmonics, and transients to simulate realistic power system conditions. The paper in [8] investigates the use of combined transducers, particularly Rogowski coils, for measuring distorted currents in electrical substations. The study shows that Rogowski coils can ensure high accuracy for harmonics up to the 50th order when used without signal conditioners. Additionally, the authors emphasize the importance of wideband accuracy classification (e.g., 0.1-WB1 class of the standard IEC 61869-1) to ensure reliable performance across frequencies up to 3000 Hz [15]. Overall, the findings stress the need to carefully consider both the transducer and associated signal conditioning for precise current metering under non-sinusoidal conditions. The article in [9] investigates the performance of iCTs under distorted current waveforms collected from the power network. The total harmonic distortion levels were up to 10%, and two commercially available iCTs were tested. The results demonstrate that the iCTs maintained their accuracy class (0.2 and 0.5) even under distorted conditions. The work in [10] introduces a method to enhance low-cost supplying test systems of instrument transformers, typically composed of a power amplifier and signal generator, by using the frequency-domain error feedback. This system enables the generation of multitone waveforms for characterizing the accuracy of distorted primary signals. In the paper in [11], a method is proposed to be adopted as the accuracy figure of the complex plane where the actual harmonic phasor is likely to be for a given confidence level. This article introduces an approach based on the Volterra model of the iCT for periodic primary current waveforms, recreating those typically found in distribution grids. The paper in [12] presents a set of accuracy verification tests for instrument transformers designed to evaluate their performance under power quality conditions characterized by harmonic and interharmonic distortion, dynamic load variations, and non-sinusoidal waveforms. These tests involve injecting synthesized distorted signals with defined harmonic and interharmonic content and transients, then comparing their output signal against a reference transducer to quantify ratio error, phase displacement, and transient response accuracy. The paper in [13] presents the development of a window-type iCT rated 400/5/1 A designed to maintain the limits of current error and phase displacement defined in the standard IEC 61869-2 for the 0.2S accuracy class across the frequency range from 50 Hz to 5 kHz [16]. Extensive experimental testing was conducted by applying sinusoidal and distorted primary currents (main 50 Hz frequency component and single higher harmonic) across the frequency range, with the iCT’s secondary current and phase displacement measured under various load conditions. The paper in [14] explores the performance of a developed iCT designed to comply with the 0.1-WB2 accuracy class of the standard IEC 61869-1 for frequencies from 50 Hz up to 20 kHz. The values of current error and phase displacement did not exceed ±0.03%/±0.05° for conditions of the load of its secondary winding resulting from the current measuring channel of the used power meter and two 2.5 m connection wires. It gives guidance on the appropriate construction of iCTs for wideband operation and the accurate transformation of distorted currents.

The investigation of the influence of primary waveform distortion on magnetic flux density in the magnetic core of iCTs is crucial, as the nonlinear B-H curve of their magnetic core causes its permeability and active power losses to change with the maximum value of the magnetic flux density [13,14,17,18,19,20,21]. Both mentioned aspects are leading to changes in iCTs transformation accuracy for different shapes of distorted current, even when its RMS value remains constant. Therefore, knowing and understanding this phenomenon is essential for proper evaluation and successful improvement of their exploitation performance under non-ideal conditions required in order to ensure accurate power metering and quality evaluation in the power system. This paper presents an analytical investigation into the influence of primary current waveform distortion on the magnetic flux density within the magnetic core of an inductive current transformer. While maintaining a constant RMS value of the primary current, it is examined how variations in the RMS values of individual harmonic components and the phase angles of the low-order higher harmonics affect the resulting magnetic flux density in the core. To investigate the observed phenomena, the waveforms of the distorted current and the corresponding magnetic flux density, as well as the resulting hysteresis loops, are presented. The analysis identifies the conditions under which, in the magnetic core of the inductive current transformer, the lowest and highest values of the maximum magnetic flux density are obtained. The study emphasizes the significant impact of the low-order higher harmonics, while demonstrating the decreasing influence of harmonic components as their frequency increases. It should be noted that greater nonlinearity of the core’s magnetization curve enhances the sensitivity of the inductive instrument transformer to changes in the magnetic flux density (also caused by the waveform distortion) and changes in the transformation accuracy of distorted current. Consequently, it is more significant to test its transformation accuracy for specific exploitation conditions resulting mainly from the load impedance of the secondary winding and the harmonic content of the primary current waveform (concerning both the RMS values of individual harmonics and their phase angles). Presented simulation results from MATLAB Simulink R2025a are verified by appropriate measurements performed in rated ampere-turns conditions of tested iCT [8,13,14,20]. Furthermore, the paper introduces realistic and worst-case test conditions based on distorted waveforms that produce maximum flux density, enabling a more accurate determination of the full range of transformation error. These contributions, supported by analytical, simulation, and experimental results, fill a critical gap in prior literature and provide a robust basis for improving iCT testing procedures under non-ideal waveform conditions.

## 2. Method and Conditions of Simulation Studies

To perform this test on 250 A/5 A iCT, an auxiliary winding with 50 turns was used, carrying a rated RMS current of 5 A. This configuration is equivalent to normal operating conditions in which a single primary conductor carrying 250 A passes through the magnetic core window. The principles of operation of the iCT and the equivalent circuit are presented, e.g., in the papers in [13,14]. The following dependence allows for the calculation of the secondary current:

(1) $$i_{2}=i_{1}-i_{0}$$

where i_1_/i_2_/i_0_—instantaneous primary/secondary/excitation current of iCT.

Consequently, it is the non-ideal magnetic core that causes transformation errors in the iCT. The following dependence allows for the calculation of the maximum value of the magnetic flux density in its magnetic core:

(2) $$B_{m}=\;max\{\frac{1}{z_{2}*s_{Fe}}\int u_{2}\left(t\right)dt\}$$

where u_2_—instantaneous secondary voltage of iCT, s_Fe_—cross section area of the magnetic core, z_2_—number of turns of the secondary winding.

The above dependence indicates that the maximum value of the secondary voltage *u*_2_ determines the maximum value of the magnetic flux density in the magnetic core. Therefore, it results from the maximum value (waveform) of the primary current and secondary winging load.

**Figure 1:**
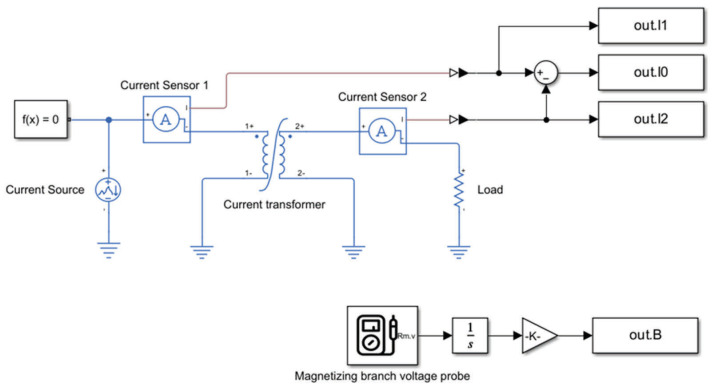
Schematic diagram of simulation circuit in MATLAB Simulink.

To investigate the impact of current harmonics on current transformer errors and operating parameters, a MATLAB Simulink model was created, utilizing the Simscape Electrical package. The model consists of a piecewise linear current source, a nonlinear transformer, and a resistor used as a load (Figure 1).

To measure the waveforms of the primary and secondary currents, two current sensors were used. The FFT analysis was performed on the obtained signal current, and angle errors were calculated for each investigated harmonic. The value of the magnetizing current was derived as a difference between the primary and secondary currents. For the purpose of the core flux density verification, the voltage across the magnetization branch of the current transformer was measured and integrated to obtain the waveform of magnetic flux and therefore the magnetic flux density. The current waveform generated by the piecewise linear current source is defined by the equation [22]:

(3) $$i\left(t\right)=\left(1-e^{-\frac{t}{\tau }}\right)\sum_{h=i}^{H}\sqrt{2}I_{h}\sin\left(\omega_{h}t+\varphi_{h}\right)$$

where t—time, τ—time constant, h—harmonic order, I_h—_RMS current of h-order harmonic, ω_h_—angular frequency of h-order harmonic, φ_h_—phase angle of h-order harmonic.

The asymptotically rising exponential component was introduced to ensure that the waveform will start at zero and to avoid the transient phenomena caused by the discontinuous change in current flowing through the inductive components.

All of the parameters used in the simulation circuit of iCT are gathered in Table 1.

The nonlinear transformer model component is based on the traditional equivalent circuit of the transformer with a nonlinear magnetizing branch [23]. The primary and secondary winding resistance was calculated based on the length of the copper wire, its cross-section, and a resistivity of 58.5 MS/m. The leakage inductance was obtained using the method described in [24], based on the real dimensions of the iCT windings and magnetic core. Since the ampere-turn method is used, both primary and secondary windings had very similar dimensions, it was assumed that the leakage inductance would be equally divided among the primary and secondary windings.

**Figure 2:**
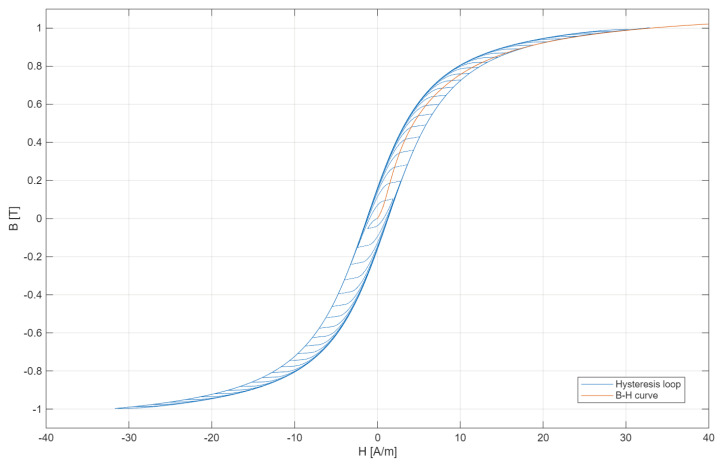
Calculated hysteresis loops and obtained B-H curve.

The magnetizing branch of the current transformer is modeled as a parallelly connected linear resistor and a nonlinear inductor. Since the CT error strongly depends on both resistive and inductive components of the magnetizing current, the nonlinearity of core losses and their dependence on flux density and frequency need to be included. For this reason, the magnetic hysteresis of the core was modeled on the basis of the Jiles–Atherton (JA) model [25,26]. The calculated hysteresis loops and obtained B-H curve are presented in Figure 2.

Since the active power losses were included in the nonlinear inductor with magnetic hysteresis, the value of the shunt resistance in the magnetizing branch was omitted by setting its value to a very large number (thousands of GΩ). In this model, the magnetic properties of the material are described by five parameters: α—interdomain coupling factor, a—domain walls density, Ms—saturation magnetization, k—average energy required to break pinnig site (bulk coupling coefficient), c—coefficient of reversible magnetization.

Parameters a and Ms are calculated automatically by the Simscape element based on the anhysteretic gradient of the B-H curve when the H is zero and the B1 (H1) point from the anhysteretic B-H curve. In the investigated case, all of the JA model’s parameters were selected manually to adjust the hysteresis curve shape to the B-H curve of the nanocrystalline core material provided by the supplier and to obtain similar core losses.

## 3. Measurement Method and Setup Used for Verification of Simulation Results

**Figure 3:**
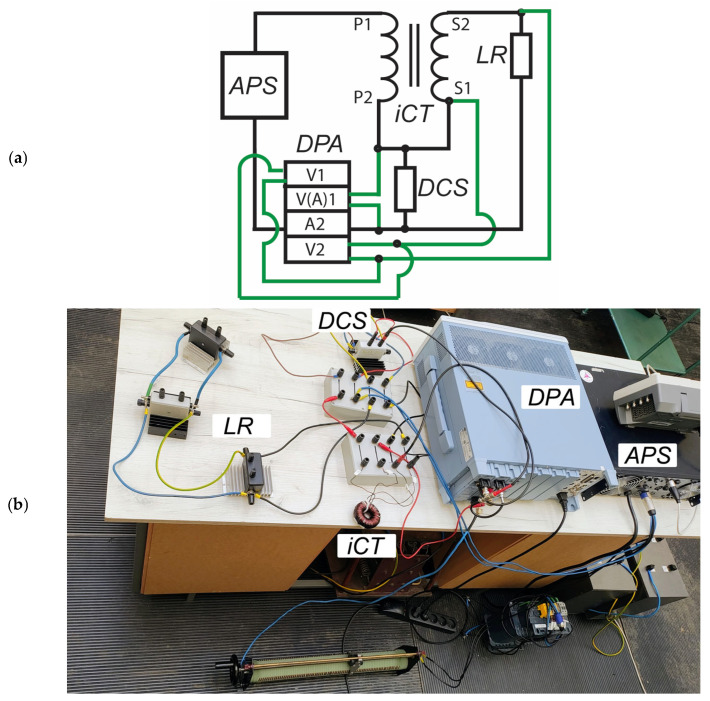
The measurement setup used for verification of simulation results: (**a**) simplified electrical diagram and (**b**) photo.

The measurement setup used for verification of simulation results is shown in Figure 3.

In Figure 3, the following notations are used: APS—arbitrary power supply, iCT—tested inductive current transformer, DPA—digital power analyzer (Yokogawa WT 5000 [27]), DCS—0.1 Ω current shunt (R_R_) used to measure the differential current between auxiliary and secondary windings, LR—load resistors of iCT’s secondary winding for 5 W and 10 W (power factor 1—noninductive resistors were used).

An arbitrary waveform generator was used for the generation of the required waveform of distorted voltage. Evaluation of iCTs transformation accuracy is performed for conditions defined for simulation. The digital power analyzer was used to simultaneously measure the RMS values of the harmonics of the current in the additional primary winding and in the differential connection between the additional primary winding and the secondary winding of the tested iCT (current shunt DCS). Moreover, the phase angles between a given order harmonics of these currents were measured in relation to the secondary voltage of tested iCT (this also enables the ability to control the secondary winding load).

The composite error value for the transformation of the h-order distorted current harmonic by the tested iCT was determined using the following equation:

(4) $$\varepsilon_{I}=\frac{U_{Rh}}{R_{R}\cdot I_{Ph}}\cdot 100\%$$

where *R_R_*—resistance of current shunt DCS, *U_Rh_—*RMS value of the h-order harmonic of voltage from current shunt DCS, *I_Ph_—*RMS value of the h-order harmonic of primary current.

The h-order distorted current harmonic in the secondary winding of the tested iCT was determined using the following equation:

(5) $$I_{Sh}=\sqrt{{I_{Ph}}^{2}+{\varepsilon_{I}}^{2}-2\cdot I_{Ph}\cdot \varepsilon_{I}\cdot \cos\theta_{h}}$$

where *θ_h_*—phase angle between the h-order harmonic of voltage from current shunt *R_R_* and primary current.

The current error value was determined using the following equation:

(6) $${∆I}_{h}=\frac{I_{Sh}-I_{Ph}}{I_{Ph}}\cdot 100\%$$

The phase displacement value in the differential measuring setup was determined using the following equation:

(7) $${\delta \varphi }_{h}=arcsin\left(\frac{\sqrt{{\varepsilon_{I}}^{2}-\Delta I_{h}^{2}}}{100\%}\right)$$

The WT5000 Digital Power Analyzer by Yokogawa Japan, recognized as the most accurate power meter currently available, was employed in this study to ensure high-precision electrical measurements. For fundamental frequency measurements within the range of 45 Hz to 66 Hz—including the nominal 50 Hz power frequency—the specified measurement accuracies for both voltage and current are ±(0.01% of reading + 0.02% of range). In the context of harmonic analysis, specifically for the 2nd to 7th harmonics (i.e., 100 Hz to 350 Hz), the measurement accuracies are ±(0.03% of reading + 0.04% of range), with an additional harmonic accuracy component of ±(0.01% of reading + 0.03% of range) applied to both voltage and current channels. These precision levels enable reliable measurements of both the fundamental and low-order harmonic components, which are critical for evaluating power quality and performance in 50 Hz electrical systems. A differential measurement system was utilized, which plays a crucial role in minimizing common-mode errors. Consequently, the composite error is directly obtained from the voltage measured on the DCS (refer to Figure 3). The expanded measurement uncertainty (U_e_) for the composite error is ±1% of reading, assuming that the measured signal is typically 25 times smaller than the measurement range (as is standard for high-accuracy iCT) and that the accuracy of the measurement resistor is also validated via analogous methods as results from Equation (4). For harmonic components up to the 7th order, the expanded uncertainty (U_e_) increases to ±3% of reading, accounting for both the resistor’s accuracy and the harmonic distortion. In accordance with Equation (5), the current in the auxiliary winding (with 50 turns) is also measured, as well as the phase angle between this current and the corresponding voltage harmonic on the DCS. However, given the use of a differential measurement system, the phase angle measurements often yield high values, but with relatively low accuracy requirements. In this context, the expanded measurement uncertainty for the secondary current is estimated at ±5% of reading, incorporating uncertainties from composite error, current measurement, and phase angle measurement (noting that power measurement uncertainty is effectively doubled due to contributions from both voltage and current). Accordingly, based on Equation (6), the U_e_ of the current error is ±6% of reading, and for higher harmonics (up to 350 Hz), this increases to ±20% of reading. It is important to note that, because a differential measurement system is used, these values refer to percentages of the current error, which itself is typically a small value expressed as a percentage. For example, if the current error for the fundamental component is 0.065%, then the corresponding expanded uncertainty is less than 0.005%, yielding a total value of 0.065% ± 0.005%. In the case of phase displacement, the expanded uncertainty is doubled, reaching approximately ±0.01°. It is important to emphasize that the value of the composite error is always considered equal to the greater of either the current error (assuming a worst-case scenario with 0° phase displacement) or the phase displacement error (converted via Equation (7), assuming zero current error). This treatment ensures a conservative and robust error estimate and illustrates the advantage of the differential measurement approach, which inherently suppresses common-mode noise and improves the reliability of the composite error evaluation.

## 4. Simulation Results with Measurement Verification

**Figure 4:**
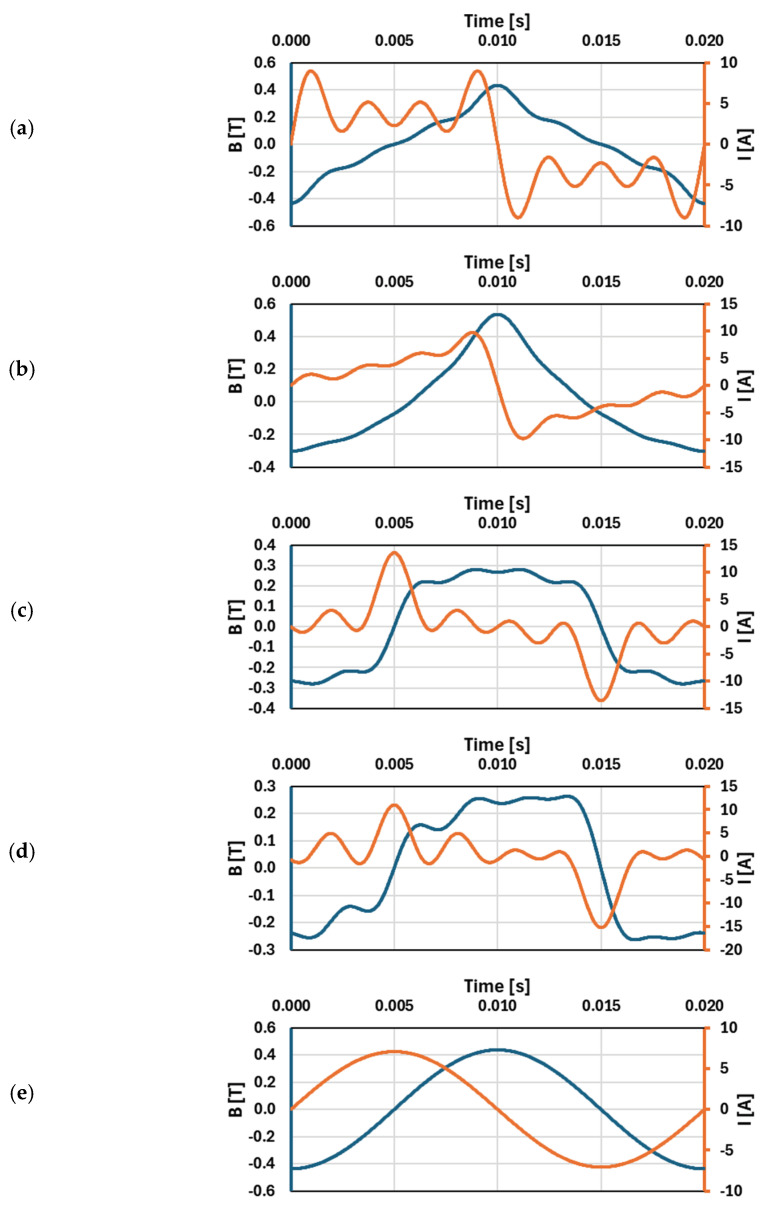
Magnetic flux density waveforms calculated in Excel, corresponding to a given primary current waveform of tested iCT, with a 5 W load on its secondary winding: (**a**) 3rd, 5th, and 7th higher harmonics; (**b**) from 2nd to 7th higher harmonics—the highest increased maximum value of the magnetic flux density; (**c**) 3rd, 5th, and 7th higher harmonics; (**d**) from 2nd to 7th higher harmonics—the lowest decreased maximum value of the magnetic flux density; and (**e**) reference sinusoidal current.

Keeping the primary current’s RMS value constant and equal to 5 A, Figure 4 presents the magnetic flux density waveforms calculated in Excel, corresponding to a given primary current waveform of tested iCT, with a 5 W load on its secondary winding:

(a) RMS values and phase angles of 3rd, 5th, and 7th higher harmonics, set in order to ensure the highest increased maximum value of the magnetic flux density in relation to the maximum value obtained in the magnetic core for sinusoidal current.
(b) RMS values and phase angles of 2nd, 3rd, 4th, 5th, 6th, and 7th higher harmonics, set in order to ensure the highest increased maximum value of the magnetic flux density.
(c) RMS values and phase angles of 3rd, 5th, and 7th higher harmonics, set in order to ensure the lowest decreased maximum value of the magnetic flux density in relation to the maximum value obtained in the magnetic core for sinusoidal current.
(d) RMS values and phase angles of 2nd, 3rd, 4th, 5th, 6th, and 7th higher harmonics, set in order to ensure the lowest decreased maximum value of the magnetic flux density.
(e) Reference sinusoidal current.

In Table 2, the RMS values and phase angles of harmonics for each waveform from Figure 4 are presented.

While maintaining a constant RMS value of the primary current equal to 5 A, it is examined how variations in the RMS values of individual harmonic components and the phase angles of the low-order higher harmonics affect the resulting magnetic flux density in the core. The analysis identifies the conditions under which, in the magnetic core of the inductive current transformer, the lowest and the highest values of the maximum magnetic flux density are obtained. In case (a), the increased maximum value of the magnetic flux density in relation to the sinusoidal current is obtained using only 3rd, 5th, and 7th higher harmonics, while in case (b), 2nd, 4th, and 6th higher harmonics are added to the primary current waveform to increase further the resulting maximum value of the magnetic flux density. The B waveform is non-symmetrical on the vertical axis due to the effect of the 2nd, 4th, and 6th low-order higher harmonics (even harmonics). In case (c), the decreased maximum value of the magnetic flux density in relation to the sinusoidal current is obtained using only 3rd, 5th, and 7th higher harmonics, while in case (d), 2nd, 4th, and 6th higher harmonics are added to the primary current waveform to further decrease the resulting maximum value of the magnetic flux density. This study emphasizes the significant impact of the low-order higher harmonics and the shape of the primary current on the exploitation conditions of iCT.

**Figure 5:**
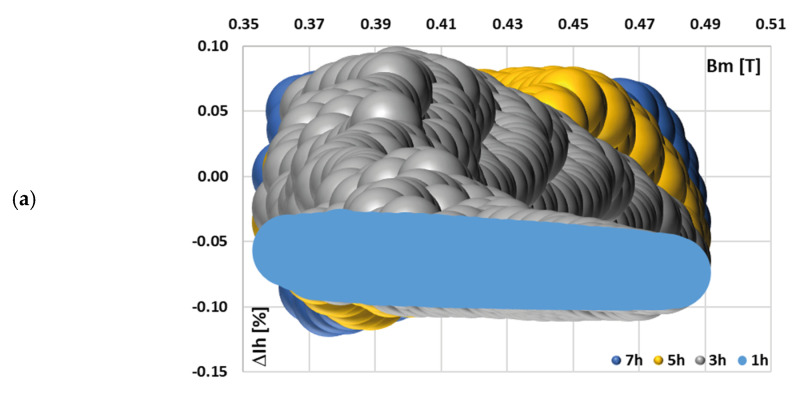

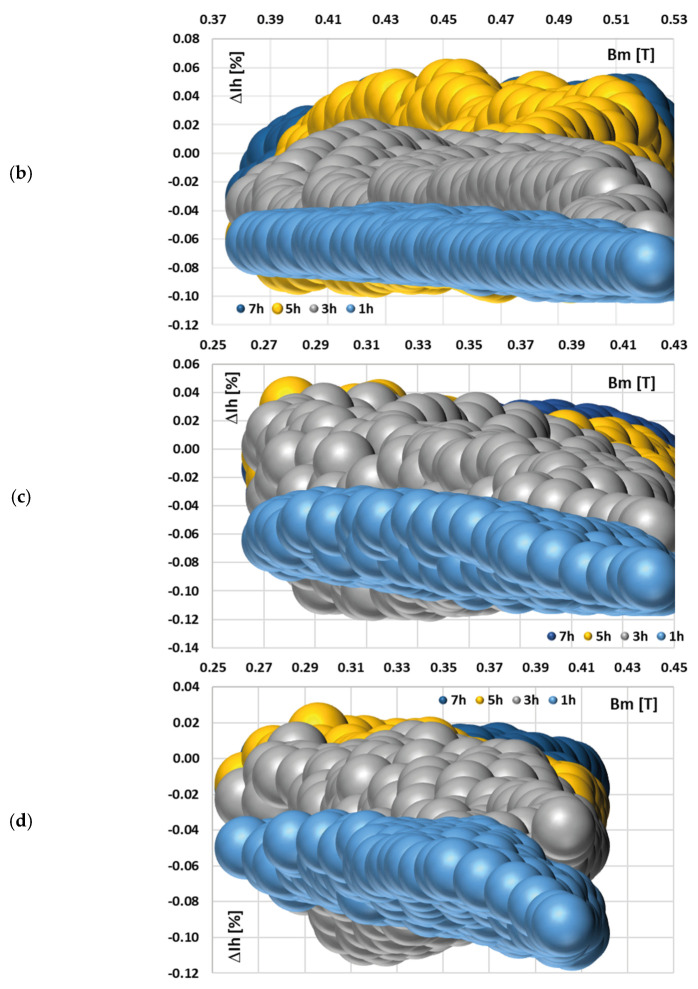
Calculated current error values for each harmonic component transformed by tested iCT with the 0.2 Ω secondary burden, obtained for the distorted current waveforms in Figure 4 and the data set from (a) to (d) in Table 2.

Figure 5 depicts the calculated current error values for each harmonic component transformed by tested iCT with a 0.2 Ω secondary burden, obtained for the distorted current waveforms in Figure 4 and the data set in Table 2.

A change in the harmonic phase angles for different RMS values of each individual harmonic within a distorted current waveform (while maintaining a constant total RMS value) resulted in a similar range of variation in the magnetic flux density (about 0.15 T) within the magnetic core. Moreover, in cases (c) and (d)—reduced maximum value of the magnetic flux density in relation to the one obtained for a sinusoidal waveform—an increase in the current error in relation to other test scenarios for the main component of distorted current is observed at the highest obtained maximum values of the magnetic flux density. Moreover, the current error determined for the 3rd higher harmonic is also slightly higher, but at the intermediate level of the maximum magnetic flux density. This is due to the fact that the magnetic properties of this core are less favorable at decreased values within this range, with the maximum value of the magnetic flux density in the magnetic core. Meanwhile, in case (a), the highest variation in current error values (from −0.09% to 0.08%) is observed. The magnetization curve of the tested iCT is quasi-linear within the range of variations in the value of the magnetic flux density. Therefore, the distribution of current error at harmonics is quite uniform for a given current waveform, with the obtained range of the change in the maximum magnetic flux density. These results highlight the critical role of harmonics phase angles, as they affect the value of current error—for different shapes of the primary current waveform, different values of current error at a given harmonics will be determined.

**Figure 6:**
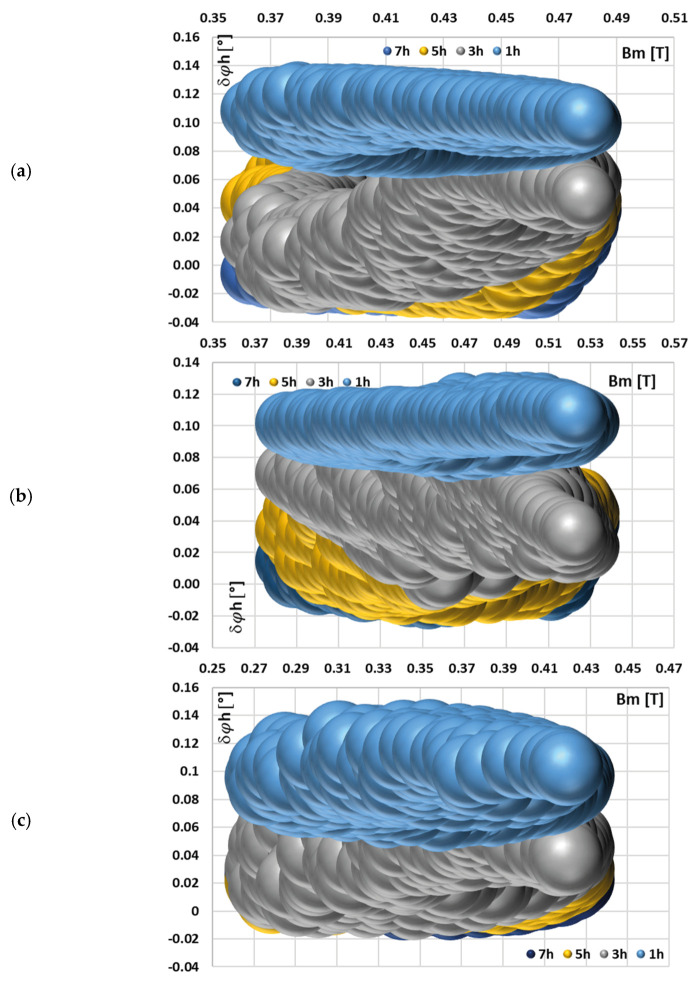

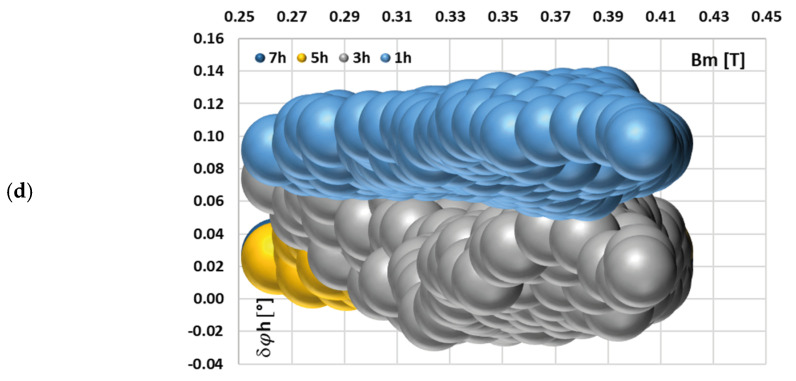
Calculated phase displacement values for each harmonic component transformed by tested iCT with the 0.2 Ω secondary burden, obtained for the distorted current waveforms in Figure 4 and the data set from (a) to (d) in Table 2.

Figure 6 depicts the calculated phase displacement values for each harmonic component transformed by tested iCT with the 0.2 Ω secondary burden, obtained for the distorted current waveforms in Figure 4 and the data set in Table 2.

A change in the maximum magnetic flux density caused by the harmonic phase angles for each waveform resulted in a similar, quite uniform distribution of phase displacement for a given harmonic of distorted current. The magnetization curve of the tested iCT is quasi-linear within the range of the variations in the value of the magnetic flux density. Therefore, the variation in harmonic phase angles for each waveform and a given higher harmonic caused a similar change in the phase displacement.

**Figure 7:**
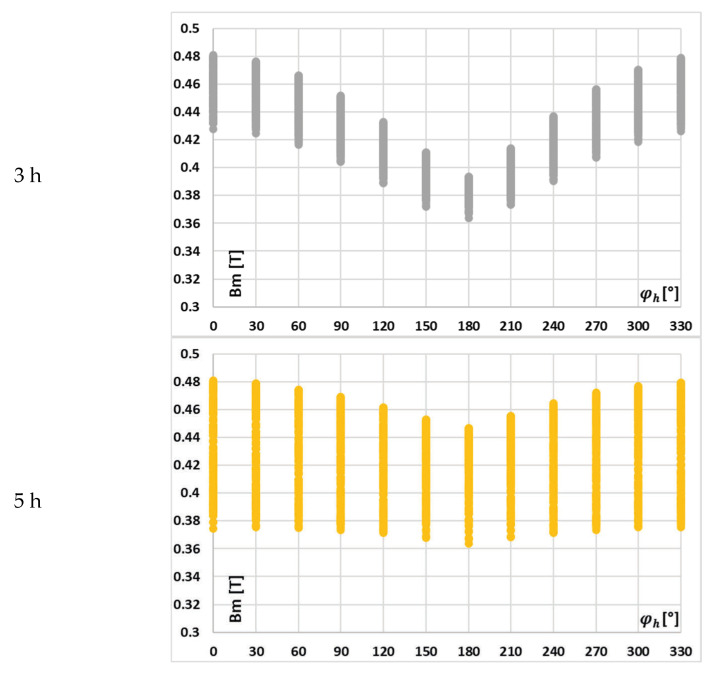

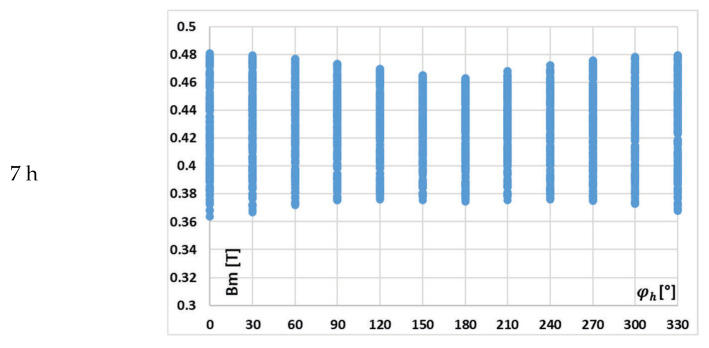
Change in the value of the maximum magnetic flux density with the phase angle of a given harmonic for waveform (a) in Table 2.

In Figure 7, the change in the value of the maximum magnetic flux density with the phase angle of a given harmonic, on the example of waveform (a) in Table 2, is presented.

It can be seen from Figure 7 that with the increase in the order of transformed higher harmonics, its influence on the value of the maximum magnetic flux density is reduced, while the effect of other harmonic phase angles becomes more important.

**Figure 8:**
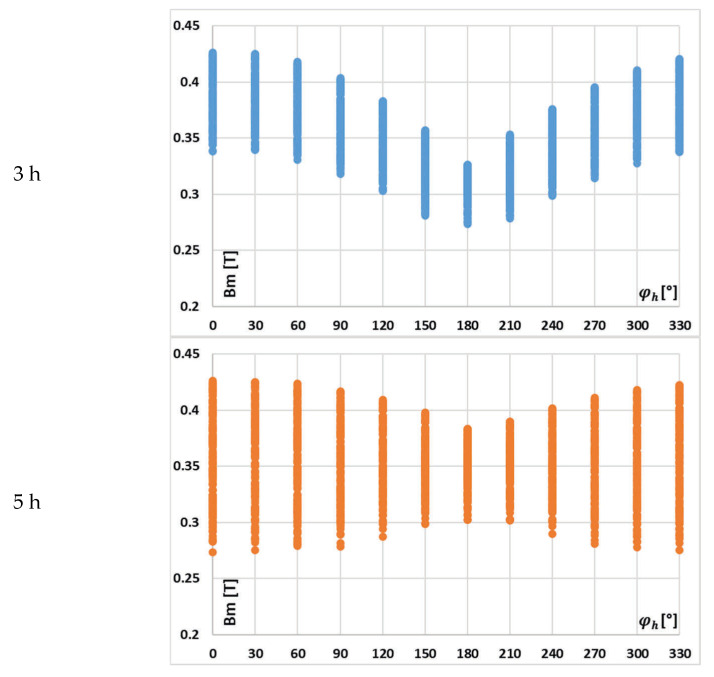

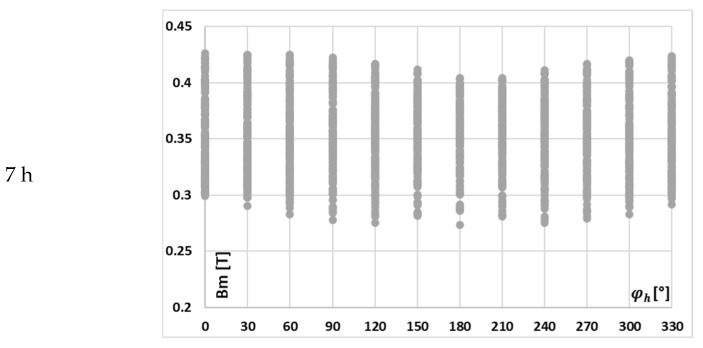
Change in the value of the maximum magnetic flux density with the phase angle of a given harmonic on the example of waveform (c) in Table 2.

In Figure 8, the change in the value of the maximum magnetic flux density with the phase angle of a given harmonic on the example of waveform (c) in Table 2 is presented.

In this case, the implications are the same as previously, and in addition, for this scenario, while the value of the maximum magnetic flux density in the magnetic core is reduced in relation to the scenario presented in Figure 7, the change in the value of the maximum magnetic flux density caused by each higher harmonics and the effect of other harmonics phase angles is greater. This is due to the fact that the RMS values of the low-order higher harmonics in the test waveforms are higher in case (c).

**Figure 9:**
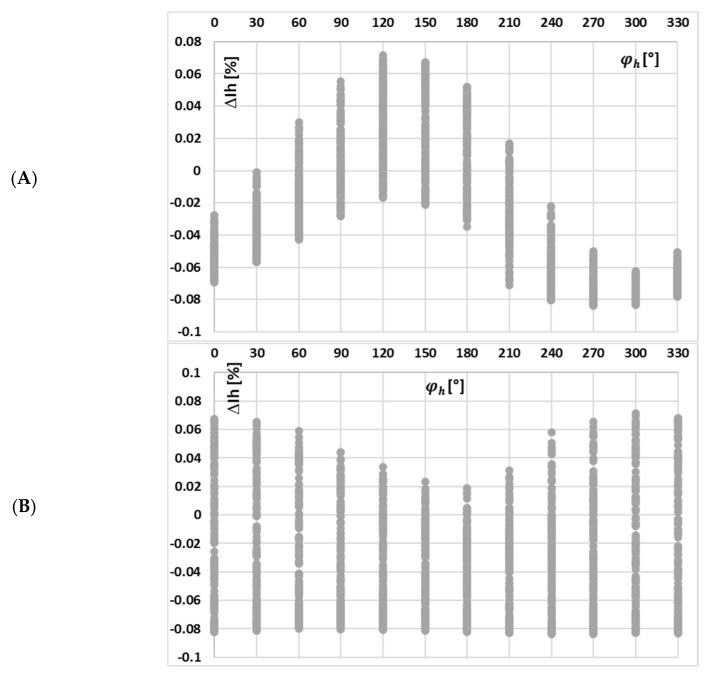

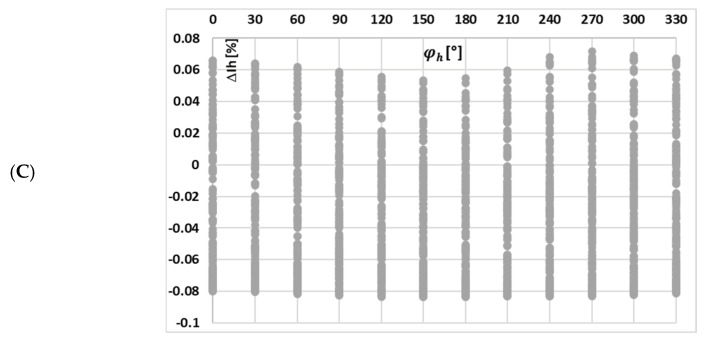
Changes in the value of current error of the 3rd low-order higher harmonic with the phase angle of higher harmonics: (**A**) 3rd, (**B**) 5th, and (**C**) 7th for the waveform (a) in Table 2.

In Figure 9, the changes in the value of current error of the 3rd low-order higher harmonic with the phase angle of higher harmonics, (A) 3rd, (B) 5th, and (C) 7th for the waveform (a) in Table 2, are presented.

Figure 9 confirms that the change in the value of current error of the 3rd low-order higher harmonic with its phase angle in relation to the 1st harmonic (50 Hz component) of the distorted primary current of iCT is significant. While the influence of the phase angle of the 5th higher harmonic is still noticeable, the impact of the change in the phase angle of the 7th higher harmonic is marginal, whereas the effect of the phase angles of other harmonics becomes more significant. It is important to note that in the results shown in Figure 9A, the variation in current error with the phase angle of the transformed higher harmonic is influenced not only by changes in the maximum magnetic flux density within the iCT’s magnetic core but also by the shifting phase angle at which the 3rd harmonic interacts with the self-generated harmonic of the same frequency 150 Hz. This self-generated component arises in the iCT’s secondary current due to the nonlinear magnetization characteristics of its magnetic core [13,14,17,18,19,20,21].

**Figure 10:**
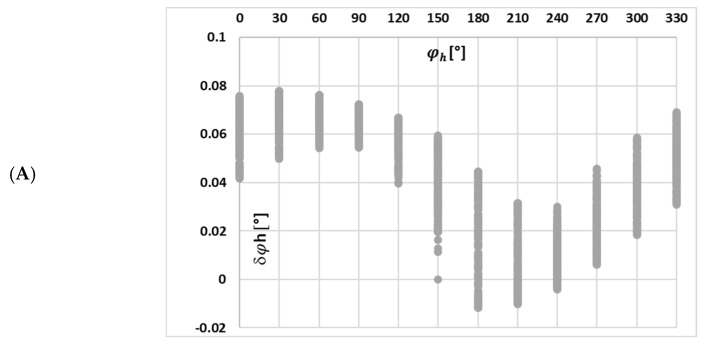

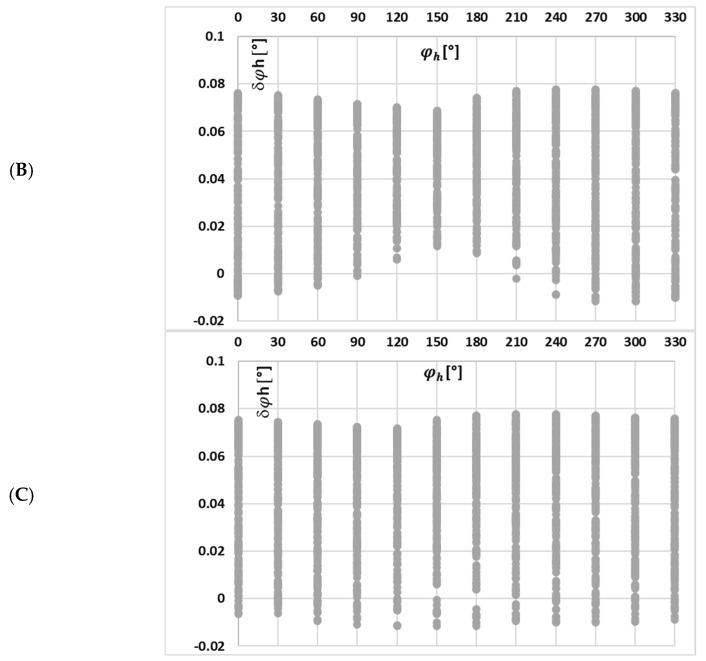
Changes in the value of phase displacement of the 3rd low-order higher harmonic with the phase angle of higher harmonic: (**A**) 3rd, (**B**) 5th, and (**C**) 7th for the waveform (a) in Table 2.

In Figure 10, the changes in the value of phase displacement of the 3rd low-order higher harmonic with the phase angle of higher harmonics, (A) 3rd, (B) 5th, and (C) 7th for the waveform (a) in Table 2, are presented.

In this case, the implications are the same as previously for the current error.

**Figure 11:**
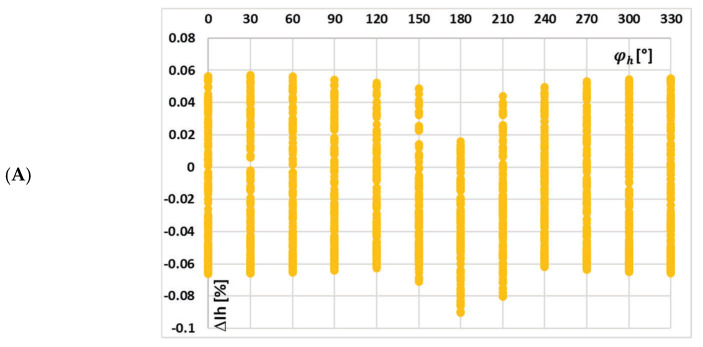

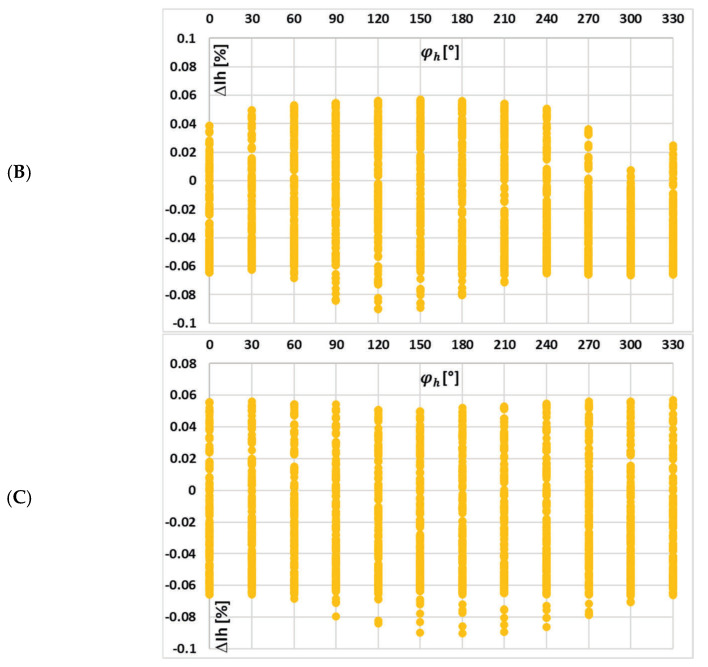
Changes in the value of current error of the 5th low-order higher harmonic with the phase angle of higher harmonic: (**A**) 3rd, (**B**) 5th, and (**C**) 7th for the waveform (a) in Table 2.

In Figure 11, the changes in the value of current error of the 5th low-order higher harmonic with the phase angle of higher harmonics, (A) 3rd, (B) 5th, and (C) 7th for the waveform (a) in Table 2, are presented.

Figure 11 demonstrates that the current error of the iCT for the 5th higher harmonic is not significantly affected by the phase angle of the 3rd low-order higher harmonic (case (A)) or by the change in its phase angle (case (B)). The effect of the 7th harmonic’s phase angle is minimal (case (C)). In contrast to the test results from Figure 9 and Figure 10, the phase angles of other harmonics play a prominent role in each case. This proves the strong influence of the low-order harmonics and the diminishing effect of higher-frequency harmonics on the magnetic flux density in the magnetic core of iCT, as well as its transformation accuracy.

**Figure 12:**
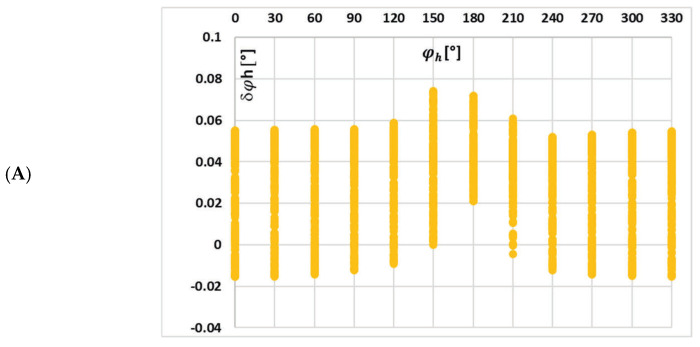

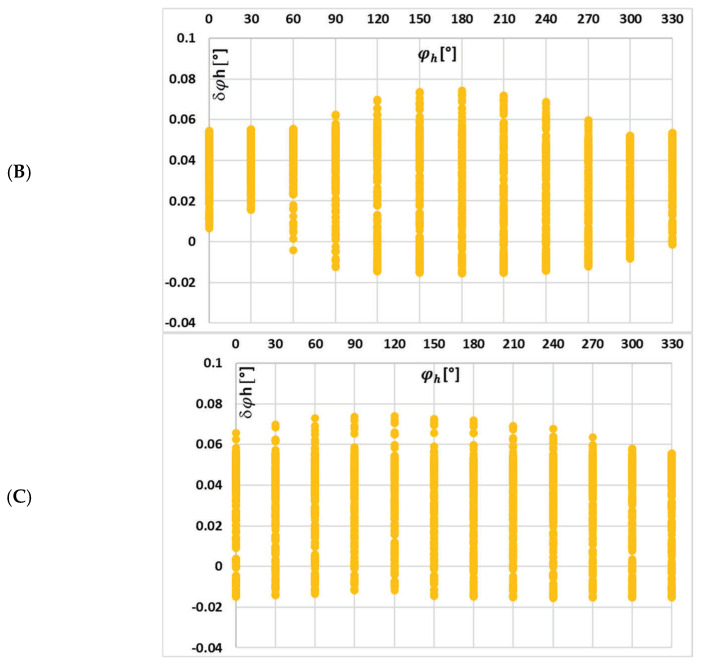
Changes in the value of phase displacement of the 5th low-order higher harmonic with the phase angle of higher harmonic: (**A**) 3rd, (**B**) 5th, and (**C**) 7th for the waveform (a) in Table 2.

In Figure 12, the changes in the value of phase displacement of the 5th low-order higher harmonic with the phase angle of higher harmonics, (A) 3rd, (B) 5th, and (C) 7th for the waveform (a) in Table 2, are presented.

In this case, the underlying mechanisms and effects are consistent with those previously discussed for the current error. Specifically, the interactions between harmonic components—particularly their phase relationships—and the nonlinear magnetic behavior of the iCT’s magnetic core continue to play a critical role in changes in its transformation accuracy of distorted current’s harmonics. This reinforces the importance of considering both harmonic content and phase angles when testing iCT performance.

It can be seen from Figure 7 and Figure 8 that with the increase in the order of transformed higher harmonics, their influence on the value of the maximum magnetic flux density is reduced. Moreover, Figure 9, Figure 10, Figure 11 and Figure 12 demonstrate that the values of current error and phase displacement of the iCT for the 3rd and 5th higher harmonics are not affected by the phase angle of the 7th low-order higher harmonic—case (C), in contrast to the test results presented in these Figures for case (A) when 3rd higher harmonic cause significantly changes. This proves the strong influence of the low-order harmonics and the diminishing effect of higher-order harmonics on the magnetic flux density in the magnetic core of iCT, as well as its transformation accuracy. Therefore, as the influence of the 7th higher harmonic was determined to be marginal, we did not consider the effect of frequency components higher than 350 Hz.

## 5. Discussion

The investigation of the influence of primary current waveform distortion on the magnetic flux density in the core of inductive current transformers (iCTs) is of critical importance. Owing to the nonlinear nature of the magnetic core characteristic, both its permeability and active power losses vary with the maximum magnetic flux density. These variations directly affect the transformation accuracy of iCTs when subjected to distorted primary currents, even when its RMS value remains unchanged. A comprehensive understanding of this phenomenon is essential for the accurate evaluation and reliable performance of iCTs under non-ideal operating conditions. Such understanding is crucial to ensure precise power metering and its quality assessment within modern power systems. This paper presents an analytical study on the impact of primary current waveform distortion on the magnetic flux density within the core of a 250 A/5 A window-type iCT, but the results are applicable to any iCT configuration. Due to the nonlinear B-H characteristic and the associated power losses, their transformation accuracy is sensitive to waveform shape—even when the RMS value of the primary current remains constant. The research demonstrates that low-order harmonics, considering both their RMS magnitudes and phase angles, substantially influence the peak magnetic flux density. Furthermore, the impact of higher-order harmonics becomes more pronounced when their RMS values are configured to shape the primary current into a rectangular waveform. These observations hold true across all types of iCTs. However, accurate calculation of specific values for current error and phase displacement requires knowledge of the simulation parameters outlined in Table 1. Unfortunately, for commercially manufactured iCTs, this information is typically unavailable and cannot be obtained through direct testing, e.g., the magnetic core material and its dimensions are not declared. In general, the current error and phase displacement characteristics of an iCT are governed by its magnetic core’s B-H curve and core loss behavior. When an increase in magnetic flux density (B) caused by the waveform harmonic content necessitates a higher magnetic field strength (H) and results in increased core losses, a decrease in transformation accuracy can be expected. This degradation in performance may be more pronounced compared with the iCT model analyzed in this study if the operating point of the iCT shifts closer to the saturation region of the B-H curve and/or if the curve exhibits greater nonlinearity. This also applies to a greater increase in active power losses with an increase in the maximum value of the magnetic flux density.

In simulations evaluating the iCT’s transformation accuracy under the influence of three harmonic components—case (a), Figure 5 and Figure 6—a total of 13,824 results were generated by varying the phase angles of each harmonic in 15-degree increments. Meanwhile, only approximately ten representative cases were selected for experimental measurement under each scenario. The measurement procedure, intended to verify simulation results, consisted of a single measurement rather than a series of repeated measurements. The current error and phase displacement values calculated using MATLAB Simulink under distorted current conditions were validated against measured data. A high level of consistency was observed for the 50 Hz fundamental component, with deviations limited to two decimal places (for example, measured current error was –0.064 percent and phase displacement was 0.11 degrees; simulated current error was –0.065 percent and phase displacement was 0.10 degrees). However, greater discrepancies were observed for higher-order harmonics. For instance, in the case of the third harmonic, the measured current error was 0.37 percent, and the phase displacement was 0.27 degrees, whereas the simulated values were 0.28 percent and 0.19 degrees, respectively. These deviations indicate that the existing MATLAB Simulink model of the iCT does not provide sufficient accuracy for harmonic distortion analysis and should be replaced with an improved model. The primary source of error appears to be associated with the modeling of magnetic hysteresis, particularly the inability to accurately reproduce the small area enclosed by the B-H hysteresis loop. To address this issue, a custom component based on a hysteresis model is currently under development. A relevant publication describing an implementation of such a model in Simulink was identified, and its reproduction yielded positive results, generating hysteresis loops consistent with those found in established references for given magnetic field intensities. However, modeling the complete transformer using this method presents significant challenges, as it requires replacing standard Simscape Electrical components with mathematical models for each element, constructed using integrators, differentiators, and algebraic loops.

The primary objective of this paper is to assess whether it is necessary to adopt a more systematic approach for testing the transformation accuracy of iCTs using distorted currents. To address this gap, this paper aims to provide evidence of the significant influence that low-order harmonics exert on transformation accuracy. While current testing protocols may rely on sinusoidal waveforms or, at best, distorted currents with a fundamental component and a single harmonic (as recommended by IEC 61869-1, derived from the now-defunct IEC 61869-6, and outlined in IEC/TR 61869-103), there remains a substantial need for a broader perspective. This paper represents an initial step toward that goal by demonstrating how simulation methods can be instrumental in determining appropriate test conditions for distorted currents. For example, if three low-order harmonics are considered, each with 24 possible phase angle values (in 15-degree increments), there are 24^3^ (13,824) unique phase combinations. If, in addition, two different RMS values are considered for each harmonic, the total number of possible test scenarios increases to 110,592. Conducting physical tests for all these combinations is impractical. However, simulations can identify the most critical conditions—those under which the greatest current error and phase displacement are likely to occur. This information is essential for developing robust testing protocols that ensure iCTs meet accuracy requirements under realistic distorted current conditions.

## 6. Conclusions

This study highlights the critical impact of primary current waveform distortion on the magnetic flux density in the magnetic core of inductive current transformers. Therefore, due to its nonlinear B-H characteristics, both permeability and active power losses vary with the peak magnetic flux density, directly affecting the transformation accuracy—even when the RMS value of the primary current remains constant. This research demonstrates that low-order harmonics, taking into consideration their RMS values and phase angles, significantly influence the resulting magnetic flux density within the magnetic core. Therefore, the change in its value caused by phase angles of higher harmonics occurs in a different range. Moreover, with the change in their phase angles in relation to the main component, the change in the value of the maximum magnetic flux density caused by each higher harmonic and the effect of other harmonic phase angles is greater if their RMS values are higher. Even harmonics regarding their phase angles may cause a further increase or decrease in the value of the maximum flux density in the magnetic core of iCT. Therefore, their impact on transformation accuracy can become significant when their RMS values are relatively high and the magnetic core exhibits strong nonlinearity in its magnetization characteristics.

To ensure reliable performance of inductive current transformers under non-ideal conditions, it is essential to assess their transformation accuracy under realistic load impedances and primary current harmonic content. During the test, it is also important to take into consideration the phase angles of higher harmonics in relation to the main component of the distorted primary current. The test conditions obtained for the distorted current waveform with the highest value of the maximum magnetic flux density ensured the determination of the highest range of the change in current error and phase displacement values for the transformation of the low-order higher harmonics. Moreover, these conditions ensure the assignment of maximum values—those that were the highest observed for the inductive current transformer across all simulated test scenarios. Even harmonics can be disregarded, as their impact on transformation accuracy was negligible, even when their RMS values were relatively high. The analytical and simulation findings, validated through experimental measurements, provide a robust foundation for understanding complexity and optimizing inductive current transformers testing for distorted currents to ensure accurate power metering and quality assessment.

## Figures and Tables

**Table 1:** Parameters used in the simulation circuit of iCT.

Parameter	Value	Unit
R_1_—primary winding resistance	0.046	Ω
R_2_—secondary winding resistance	0.040	Ω
L_1_—leakage inductance of auxiliary winding	1.057	μH
L_2_—leakage inductance of secondary winding	1.057	μH
l_Fe_—average length of magnetic flux path	150.8	mm
s_fe_—cross section area of the magnetic core	247	mm^2^
B_0_/H_0_—anhysteretic B-H gradient when H is zero	0.1	T × m/A
B_1_—flux density point on anhysteretic B-H curve	1.1	T
H_1_—field strength point on anhysteretic B-H curve	160	A/m
c—coefficient for reversible magnetization	0.1	-
k—Bulk coupling coefficient	1.45	A/m
α—interdomain coupling factor	3 × 10^−6^	-

**Table 2:** RMS values and phase angles of harmonics for each waveform from Figure 4.

Waveform	B_m_ [T]	RMS Value [A]							Phase Angle in Relation to h1 [°]					
		I_h1_	I_h2_	I_h3_	I_h4_	I_h5_	I_h6_	I_h7_	I_h2_	I_h3_	I_h4_	I_h5_	I_h6_	I_h7_
(a)	0.473	4.6	0	1.5	0	1	0	0.75	0	0	0	0	0	0
(b)	0.536	4	2	1.5	1	1	0.5	0.75	180	0	180	0	180	0
(c)	0.28	3.6	0	2	0	2	0	2	0	180	0	0	0	180
(d)	0.26	3.3	1	2	1	2	0.5	2	90	180	270	0	270	180
(e)	0.437	5	0	0	0	0	0	0	0	0	0	0	0	0

## Data Availability

Data are provided within the manuscript.
